# Complete mitochondrial genomes of the Southeast Asian freshwater pufferfishes, *Pao abei* (Roberts, 1998) and *Pao suvattii* (Sontirat and Soonthornsatit, 1985) (Tetraodontiformes: Tetraodontidae) and an insight into the taxonomic status of *Pao* species

**DOI:** 10.1080/23802359.2021.1911708

**Published:** 2021-04-20

**Authors:** Akinori Yamada, Ayaka Hamaguchi, Hikari Sakoda, Motohiro Kakamu, Hiroyuki Doi, Sasitorn Hasin, Osamu Arakawa

**Affiliations:** a Graduate School of Fisheries and Environmental Sciences, Nagasaki University, Nagasaki, Japan; b Faculty of Fisheries, Nagasaki University, Nagasaki, Japan; c Nifrel, Suita, Japan; d Innovation of Environmental Management, College of Innovative Management, Valaya Alongkorn Rajabhat University Under the Royal Patronage, Pathumthani, Thailand

**Keywords:** *Monotrete*, *Tetraodon*, Mekong river, mitogenome, Thailand

## Abstract

The complete mitochondrial genomes of the Southeast Asian freshwater pufferfishes, *Pao abei* and *Pao suvattii*, were reconstructed using the MGISEQ platform. The genomes were 16,448 bp and 16,449 bp in length, each made up of 37 mitochondrial genes (13 CDSs, 22 tRNAs, and two rRNAs) and putative control region. It is suggested that an accumulation of complete mitochondrial genome sequences can contribute to resolve the taxonomic status of *Pao* species.

The genus *Pao* is a member of the family Tetraodontidae (pufferfish). It was formerly placed in the genus *Monotrete*, for which there is currently no available name, and before that, in the genus *Tetraodon* (Kottelat 2013). According to Fricke et al. (2021), at total of 15 valid species are recognized, while the taxonomic status of these species has been repeatedly argued (Roberts 1998; Kottelat 2013; Saenjundaeng et al. 2013). They inhabit Southeast Asian freshwaters, and many of them have been recorded from the Mekong river basin flowing through Yunnan (Mainland China), Cambodia, Laos, Myanmar, Thailand, and Vietnam. Previous studies showed that several species possess a potent neurotoxin, saxitoxins (STXs), and that the toxicity would depend on the species or its accompanying genetic properties (Arakawa et al. 2017; Zhu et al. 2020). Freshwater puffers are potential food sources for local people, whereas poisoning incidents have occurred probably through an accidental consumption of toxic individuals. This could have been caused by the great variability of color pattern and the lack of distinctive morphological characters to diagnose *Pao* species. Using genetic information could greatly improve on the identification and differentiation.

Specimens of *Pao abei* (Roberts 1998) and *Pao suvattii* (Sontirat and Soonthornsatit 1985) were derived from individuals bred from wild populations located in the Chao Phraya river basin (100°31′E, 13°44′N; Bangkok) and the Mekong river basin (105°18′E, 15°42′N; Ubon Ratchathani province), respectively. The total length and weight of the *P. abei* specimen (specimen voucher: Nagasaki University #PA20190905-2) were 103 mm and 34.4 g, and those of the *P. suvattii* specimen (#PS20190905-1) were 150 mm and 111.6 g. Species of the specimens were morphologically confirmed according to Roberts (1998) and Sontirat and Soonthornsatit (1985). In particular, the *P. abei* specimen was differentiated from the other species by the presence of non-ocellated orange-colored round spots (*cf*. Roberts 1998). Total DNA was extracted from muscle, purified, and used for the whole genome shotgun libraries construction. The libraries were circularized, clonally amplified and modified to produce DNA nanoballs. A total of approximately 45 M 150-bp paired-end reads generated by DNBSEQ-G400 for each library were assembled using IDBA_UD (Peng et al. 2012). Circular contigs of the mitochondrial genome were reconstructed and manually annotated by comparing with the existing genomes in the database and by referring to the rRNA and tRNA second structure models (Satoh et al. 2016). Phylogenetic analysis using Bayesian inference was conducted using MrBayes 3.2.7 (Ronquist et al. 2012).

The complete mitochondrial genomes of *P. abei* and *P. suvattii* were 16,448 bp and 16,449 bp in length, respectively, and both contained 37 mitochondrial genes (13 CDSs, 22 tRNAs, and two rRNAs) and putative control region. The Bayesian tree based on concatenated nucleotide sequences of 13 CDSs indicated the sequences of the two specimens, *P. abei* and *P. leiurus* (KF667490.1, Hu et al. 2015), were closely related to each other (Figure 1). Their nucleotide sequence identity of the complete mitochondrial genomes was 99.98%, which is apparently within the range of a species.

**Figure 1.**
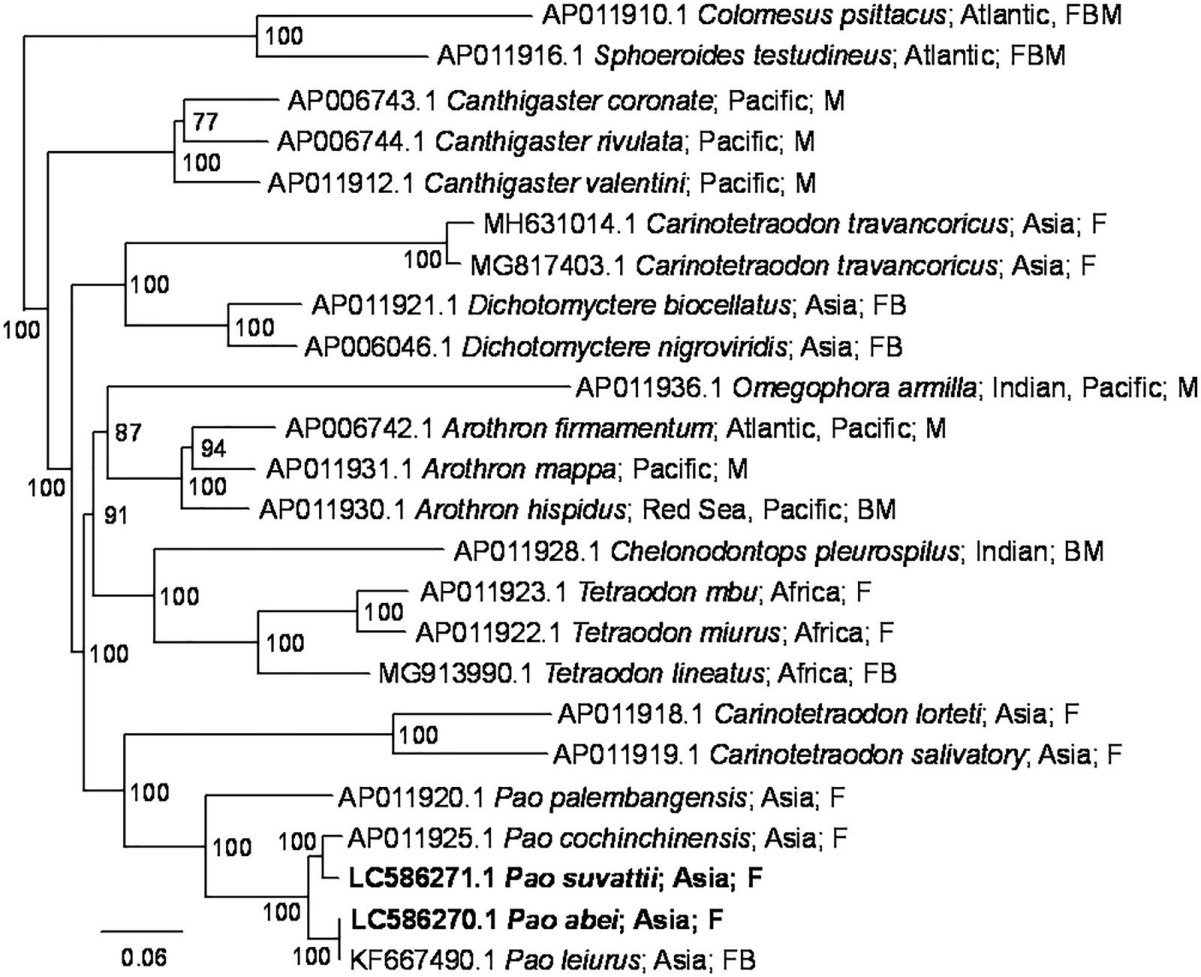
Phylogenetic relationship of *Pao* and related species inferred from concatenated nucleotide sequences of 13 CDSs using Bayesian inference. In the Bayesian analysis, the best fit model (GTR + G+I model) was selected by Kakusan4 (Tanabe 2011), 12 runs of 2.5 million generations were performed with four chains each, trees were sampled at 1000-generation intervals, and the first 10% of the trees were discarded as burn-in. The new sequences are shown in bold. Distributions and habitats (F: fresh water, B: brackish water; M: marine water) given by Fricke et al. (2021) are indicated with accession numbers and species names. Numbers at each node represent Bayesian posterior probabilities. *C. psittacus* and *S. testudineus* were used as outgroups.

Several nominal species have been frequently synonymized as *P. leiurus*, which is the oldest name of this genus, while at the same time, they have been revalidated by various authors (Roberts 1998; Kottelat 2013; Saenjundaeng et al. 2013). Although *P. abei* and *P. leiurus* are both currently valid species (Kottelat 2013; Fricke et al. 2021), that may suggest the possibility of *P. abei* being a synonym of *P. leiurus* and vice versa, or the existence of another species to which the two specimens and/or the local populations would belong. As the genus *Pao* is, on another hand, shown to have evolved relatively recently (Santini et al. 2013), several lineages may have not yet diverged sufficiently to be separated using mitochondrial sequences. Therefore, more accumulation of complete mitochondrial genome sequences with morphological features can clarify delimiting species boundaries and contribute to food safety in future.

## Data Availability

All assembled mitochondrial genomes are available on GenBank using the ascension numbers: LC586270 and LC586271.
